# Southeast Asian clearwing moths buzz like their model bees

**DOI:** 10.1186/s12983-021-00419-8

**Published:** 2021-07-07

**Authors:** Marta Skowron Volponi, Luca Pietro Casacci, Paolo Volponi, Francesca Barbero

**Affiliations:** 1 Laboratory of Evolutionary Biology and Insect Ecology, Faculty of Biology, University of Bialystok, Ciołkowskiego 1J, 15-245 Białystok, Poland; 2 ClearWing Foundation for Biodiversity, Podczaszyńskiego 11/15 m 23A, 01-866 Warsaw, Poland; 3 Department of Life Sciences and Systems Biology, University of Turin, Via Accademia Albertina 13, 10123 Torino, Italy

**Keywords:** Acoustic mimicry-aposematism-behavioural ecology-hymenopteran mimicry-predator prey interactions-Sesiidae

## Abstract

**Background:**

The endless struggle to survive has driven harmless species to evolve elaborate strategies of deceiving predators. Batesian mimicry involves imitations of noxious species’ warning signals by palatable mimics. Clearwing moths (Lepidoptera: Sesiidae), incapable of inflicting painful bites or stings, resemble bees or wasps in their morphology and sometimes imitate their behaviours. An entirely unexplored type of deception in sesiids is acoustic mimicry. We recorded the buzzing sounds of two species of Southeast Asian clearwing moths, *Heterosphecia pahangensis* and *H. hyaloptera* and compared them to their visual model bee, *Tetragonilla collina*, and two control species of bees occurring in the same habitat. Recordings were performed on untethered, flying insects in nature.

**Results:**

Based on eight acoustic parameters and wingbeat frequencies calculated from slow-motion videos, we found that the buzzes produced by both clearwing moths highly resemble those of *T. collina* but differ from the two control species of bees.

**Conclusions:**

Acoustic similarities to bees, alongside morphological and behavioural imitations, indicate that clearwing moths display multimodal mimicry of their evolutionary models.

**Supplementary Information:**

The online version contains supplementary material available at 10.1186/s12983-021-00419-8.

## Background

In the evolutionary arms race between predator and prey, hymenopterans have evolved active defence mechanisms [1]. Although bees and wasps are capable of inflicting a painful sting upon a predator, it is still more beneficial for them to avoid attack, and thus the risk of getting killed or injured in the process, by displaying warning signals [2]. Aposematism is a defence strategy whereby a potential prey advertises its unprofitability, e.g. due to its toxicity, aggressive nature, or even high energetic cost of chase and capture [3], to a predator. Aposematic signals include bright (or at least contrasting with the background) colouration, deterring sounds or early warnings such as reflex, odorous bleeding in ladybirds [4]. After engaging in an unpleasant encounter, e.g. with a bee or a wasp, predators learn to recognise their warning displays and in the abundance of other, unprotected prey, avoid attacking a hymenopteran. Harmful hymenopterans endowed with similar warning signals form complexes of Müllerian mimicry (where multiple unpalatable species benefit from having evolved mutual aposematic resemblance) in which they profit from sharing the per capita mortality during the stage of predator learning [4]. Their relationship is mutualistic, even when species forming the complex are unequally defended (e.g. some bees are able to sting, others have reduced stings but can still bite, and some visually similar species form complexes with varying levels of toxicity).

One of the crucial concepts in evolutionary biology, described initially by Bates in 1862 [5] and thus termed Batesian mimicry, states that harmless species benefit from resembling noxious species. Batesian mimics take advantage of predators’ learned avoidance, and are thus parasites which can increase attacks on their models by weakening predators’ discrimination abilities of true defence signals [6]. Bates noticed visual aspects of the phenomenon [5], however further studies revealed the existence of complex, multimodal mimicry, in which morphological resemblances go in pair with imitations of behaviour [7], sounds [8, 9] and chemical signals [10]. For instance, the parasitoid wasp *Gelis agilis* Fabricius, 1775 (Hymenoptera: Ichneumonidae) morphologically and behaviourally resembles *Lasius niger* Linnaeus, 1758 (Hymenoptera: Formicidae) ants, and releases an odorous volatile that acts as an alarm pheromone in some ant species. Thus, the wasp gains protection from predators including wolf spiders by sending multiple signals at the same time [11]. Another case of multimodal mimicry is that displayed by colubrids. These mostly non-venomous snakes, apart from visually resembling vipers, imitate a full array of their anti-predatory displays, including the production of hissing sounds and striking at the intruder [12]. Few other invertebrates have evolved more remarkable resemblances to their evolutionary models than those of the lepidopteran family Sesiidae (clearwing moths). However, due to their rarity, difficulty in locating them in the field, as well as seasonal and hard-to-predict occurrence, Southeast Asian clearwing moths are generally poorly studied, and aspects of their behaviour and biology are only now being discovered (e.g. [13–15].

Sesiids are diurnal and sometimes crepuscular moths morphologically resembling hymenopterans whose most striking feature are narrow wings, either entirely transparent or with hyaline patches. In the course of evolution, clearwing moths have strongly reduced the amount of alar scales so that they often cover only the veins and contours. Their larvae bore inside various parts of plants, remaining hidden during the preimaginal stages. In temperate regions, they are either univoltine or semivoltine, having one generation every 1–3 years. The imago is the shortest life stage, with adults typically living from several days in some species to as long as several months in others (e.g. *Synanthedon myopaeformis* Borkhausen, 1789, [16]). Presumably, tropical clearwing moths have a much shorter lifespan than temperate species, as is the case in other groups of insects. Those tropical sesiids which have been studied are mostly univoltine; however bivoltinism has been suggested for some species (e.g. by Arita & Gorbunov, 1996 [17] for members of the genus *Melittia* Hübner, 1819) and multivoltinism is likely to occur in *Heterosphecia pahangensis* Skowron, 2015 and *Heterosphecia hyaloptera* Hampson, 1919, species we focus on within this study. Recent morphological and DNA analyses have shown that *H. bantanakai* Arita & Gorbunov, 2000 is a junior synonym of *H. hyaloptera* (manuscript in preparation) and we conform to the revised nomenclature here. The generic placement of *H. pahangensis* is also going to change in the near future (manuscript in preparation).

**Fig. 1:**
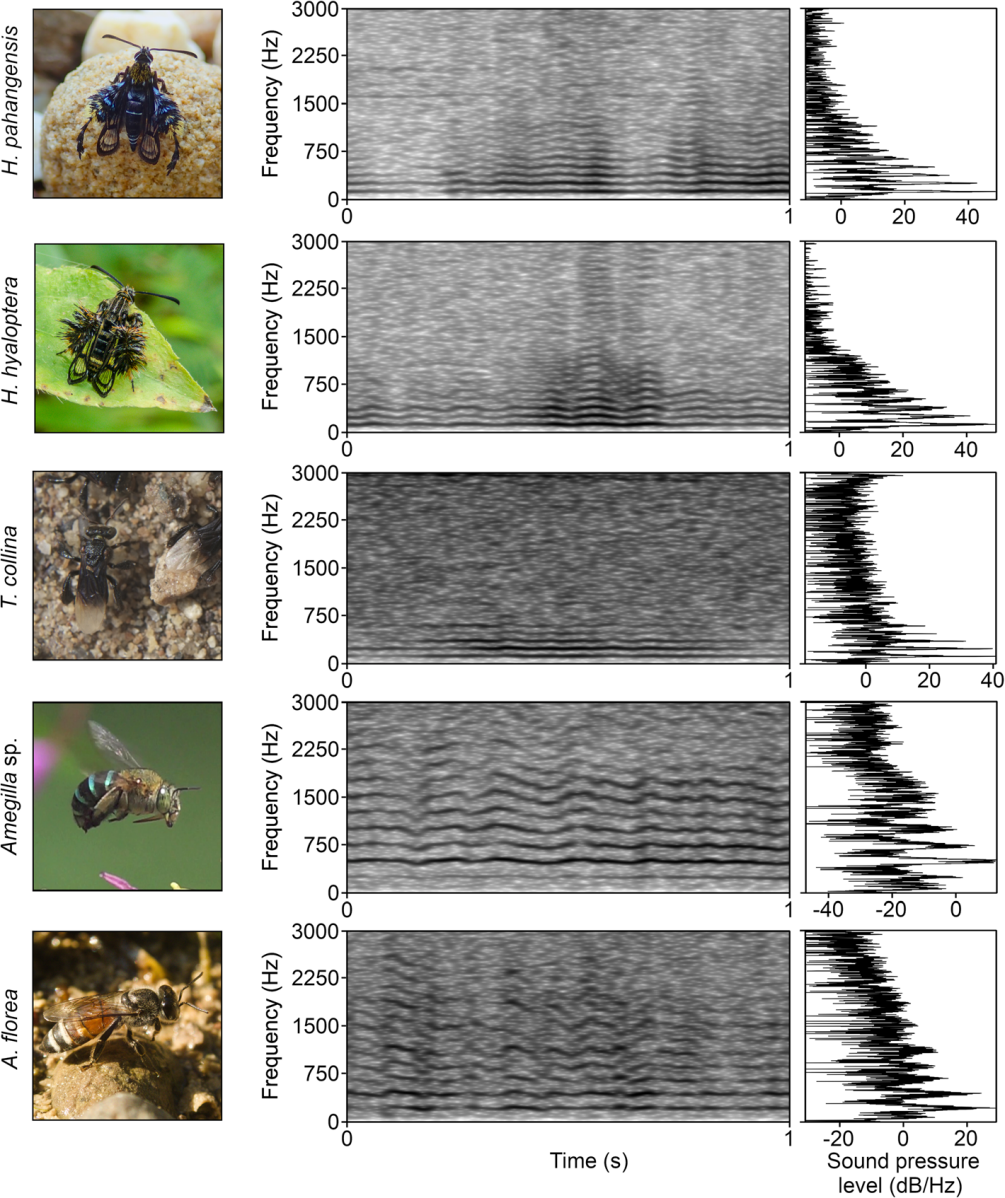
Spectrograms and FFT slices of the wing buzzing of the five recorded species. Spectrograms were generated in Praat version 6.1.05 using a Gaussian window shape, a window length of 0.05 s, 1000 time steps, 250 frequency steps and a dynamic range of 70 dB

*Heterosphecia pahangensis* is a Malaysian clearwing moth that morphologically and behaviourally resembles sympatric *Tetragonilla collina* Smith, 1857 bees. It is a small (wingspan 9–14 mm) sesiid, mostly black with almost entirely transparent wings, several narrow white bands on the abdomen and highly conspicuous tufts of elongated scales on the hind legs (Fig. 1; Additional file 1 time code 00:00–00:25, 01:03–01:28; [18]). These tufts look like specialised hairs used for gathering pollen on the hind legs of bees and are bluish-black with an addition of various amounts of yellow and white scales, which in some individuals form patches of yellow colouration resembling pollen. *H. pahangensis* puddles for salt in the same habitats as the similar-sized, black *T. collina* bees and when searching for a puddling spot, both insects fly in zigzag trajectories [15] which makes them extremely difficult to tell apart. These imitations of bee locomotion are a striking example of behavioural mimicry which reinforces predator-deceiving visual signals.


**Additional file 1**. Video showing the studied species in their habitat and slow motion shots from which wingbeat frequencies were calculated. Available at https://vimeo.com/512990904

An entirely unexplored type of deception open to exploitation by clearwing moths to deter predators is acoustic mimicry. *H. pahangensis* does indeed emit sounds when flying (Skowron Volponi & Volponi, pers. obs.). The potential ability to buzz like its model bee would benefit this harmless species by serving as a warning signal (albeit false), not only upon attack, but also to prevent them.

To test whether this clearwing moth produces acoustic signals similar to those used by its model, we recorded the sounds emitted by *H. pahangensis* in flight and compared them to the buzzing of *T. collina* and two control species of bees occurring in the same habitat in Southeast Asia: *Apis florea* Fabricius, 1786 and *Amegilla* sp. Friese, 1897. We also recorded *H. hyaloptera*, another clearwing moth of similar posture, colouration and foraging habits to *H. pahangensis* and *T. collina* (Fig. 1; Additional file 1 TC 00:26–00:58, 01:29–01:49). *H. hyaloptera* occurs in Thailand and further North up to China but is not known from Malaysia. *T. collina*, which is widespread in both Malaysia and Thailand [19] could potentially serve as a model for both studied sesiid species. *T. collina* lack functional stings and thus cannot inject venom, however they can still bite and chase away intruders [20]. Predators, especially when attacked by a group of aggressive bees defending their nest, are likely to recognise them as unpalatable, even though *T. collina* is not chemically defended. A similar system, also from the Malay archipelago, occurs in orioles that mimic friarbirds. The latter birds act aggressively when disturbed. Apparently, their aggressive nature provides enough protection from predators for natural selection to have favoured increasing morphological similarity of the gentle orioles to fierce friarbirds serving as mimicry models [21].

Despite clearwing moths being striking visual mimics of hymenopterans, the meaning of the buzzing sounds they produce has never been explained in terms of potential acoustic mimicry. Verifying whether acoustical similarities exist between two species of clearwing moths, which occur in different countries but both resemble a bee widespread in mainland Southeast Asia, will allow us to assess if acoustic mimicry is a geographically extended phenomenon or just a local adaptation of *H. pahangensis*. Overall, we hypothesise that multimodal mimicry occurs in the *H. pahangensis* (mimic) - *T. collina* (model) system.

## Results

When flying, the wing vibration of all of the studied species produces continuous, harmonic sounds (Fig. 1). The number of harmonics visible on a spectrogram varies between species, but the first three frequency components are well distinguishable in the sound spectra of all studied species. In most recordings of *H. pahangensis*, *H. hyaloptera* and *T. collina*, five frequency components (including the fundamental frequency) are visible, reaching almost 690 Hz in *H. pahangensis*, 650 Hz in *H. hyaloptera* and 640 Hz in *T. collina*. *A. florea* sounds consist of four well-defined and sometimes two more faint frequency components reaching 1380 Hz, whereas *Amegilla* sp. has 8–10 distinct and up to six weaker additional components, reaching almost 4000 Hz. Both studied species of clearwing moths and the model *T. collina* produce low-frequency sounds with the mean dominant frequency oscillating around 120–125 Hz. The two control species of bees, by contrast, have significantly higher mean dominant frequencies, just above 220 Hz in *A. florea* and 460 Hz in *Amegilla* sp. (see the Univariate analysis for details).

### Univariate analysis

**Fig. 2:**
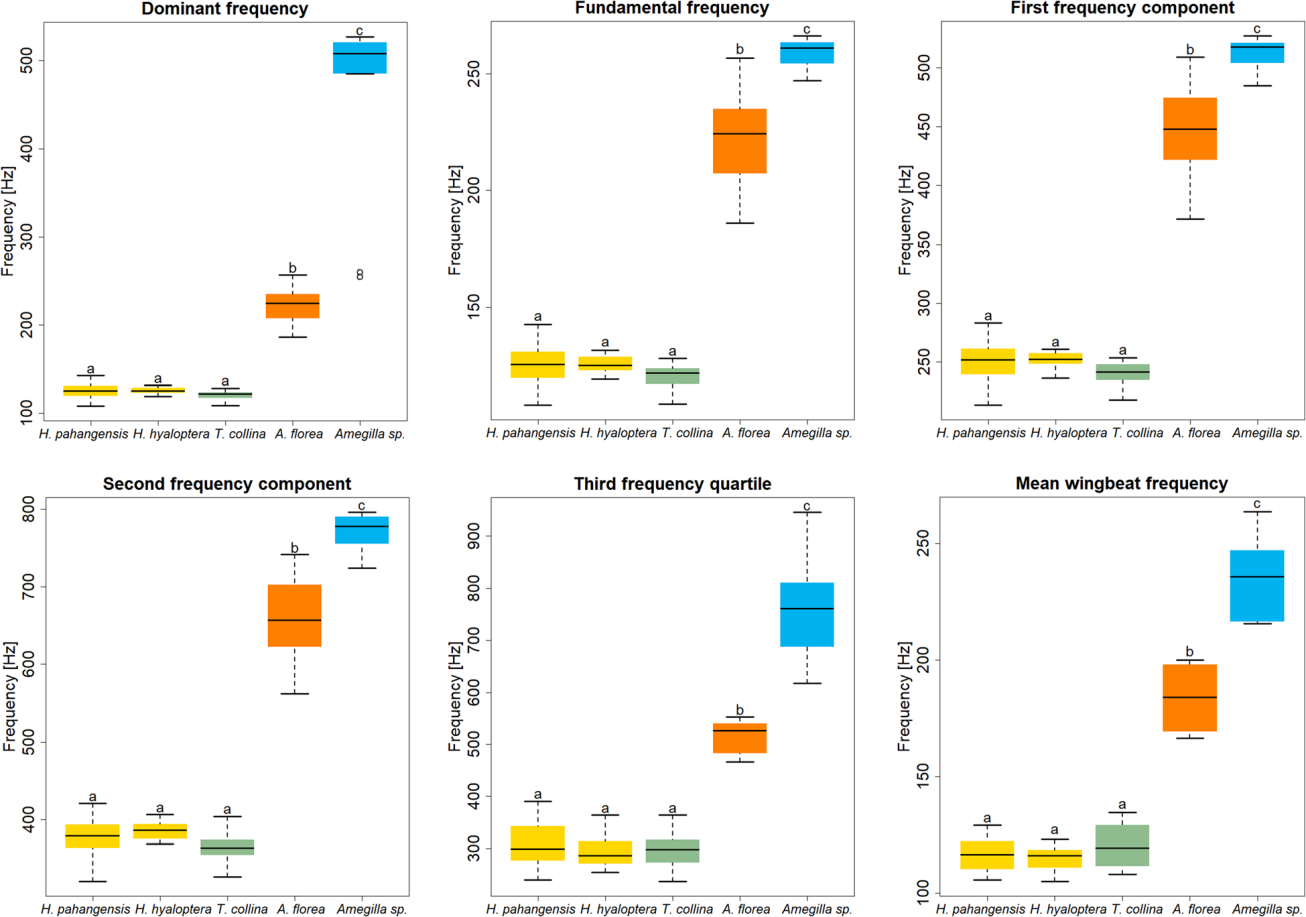
Boxplots showing differences between species for the following parameters: dominant frequency, fundamental frequency, first and second frequency components above fundamental frequency, third frequency quartile and mean wingbeat frequency (statistical tests are reported in the main text). Different letters above boxplots indicate statistically significant differences between species based on Tukey’s HSD test (for exact values see Tables 1, 2, 3, 4, 5 and 6 in Additional file 2). Open circles represent outliers

Frequency components and dominant frequency vary among the five species’ sounds (Likelihood Ratio test- LR_Fundamental Freq_. χ^2^_132,4_ = 115.13, *p* < 0.001; LR_First Freq_. χ^2^_132,4_ = 119.66, *p* < 0.001; LR_Second Freq_. χ^2^_132,4_ = 120.07, *p* < 0.001; LR_Dominant Freq_. χ^2^_132,4_ = 115.13, *p* < 0.001; Fig. 2). However, neither the frequency components nor the dominant frequency differ significantly between the buzz emitted by the two clearwing moths and the stingless bee model (values of pairwise tests are reported in Tables 1, 2, 3 and 4 in Additional file 2). In contrast, dwarf honey bees and blue banded bees, the two control species, buzz with a significantly higher dominant frequency and show differences in all frequency components compared to both clearwing moths and stingless bees (Tables 1, 2, 3 and 4 in Additional file 2; Fig. 2).

Similarly, sound parameters such as the third frequency quartile (Kruskal-Wallis χ^2^_132,4_ = 60.509, *p <* 0.001; Table 5 in Additional file 2) and standard deviation of the frequency spectrum (Kruskal-Wallis χ^2^_132,4_ = 37.525, *p* < 0.001; Table S6) vary among the five species, but do not differ between clearwing moths and stingless bees or between the two controls, *A. florea* and *Amegilla* sp.

More precisely, the standard deviation of the frequency spectrum is also similar in buzzing sounds produced by *H. pahangensis* and *A. florea*, whereas the third frequency quartile differs between all mimics, as well as model and the two controls (Fig. 2; Table 5 in Additional file 2).

### Multivariate analysis

According to correlation analysis, the three frequency quartiles are highly correlated (r = 0.96 for the first and second quartile, *r* = 0.85 for the first and third quartile and r = 0.91 for the second and third quartile) and thus only the third quartile has been kept for further analysis. The following acoustic variables are considered in PLS-DA: fundamental frequency (Hz), frequency of the first and second component (Hz), dominant frequency (Hz), third frequency quartile (Hz), standard deviation of the frequency spectrum (Hz).

**Fig. 3:**
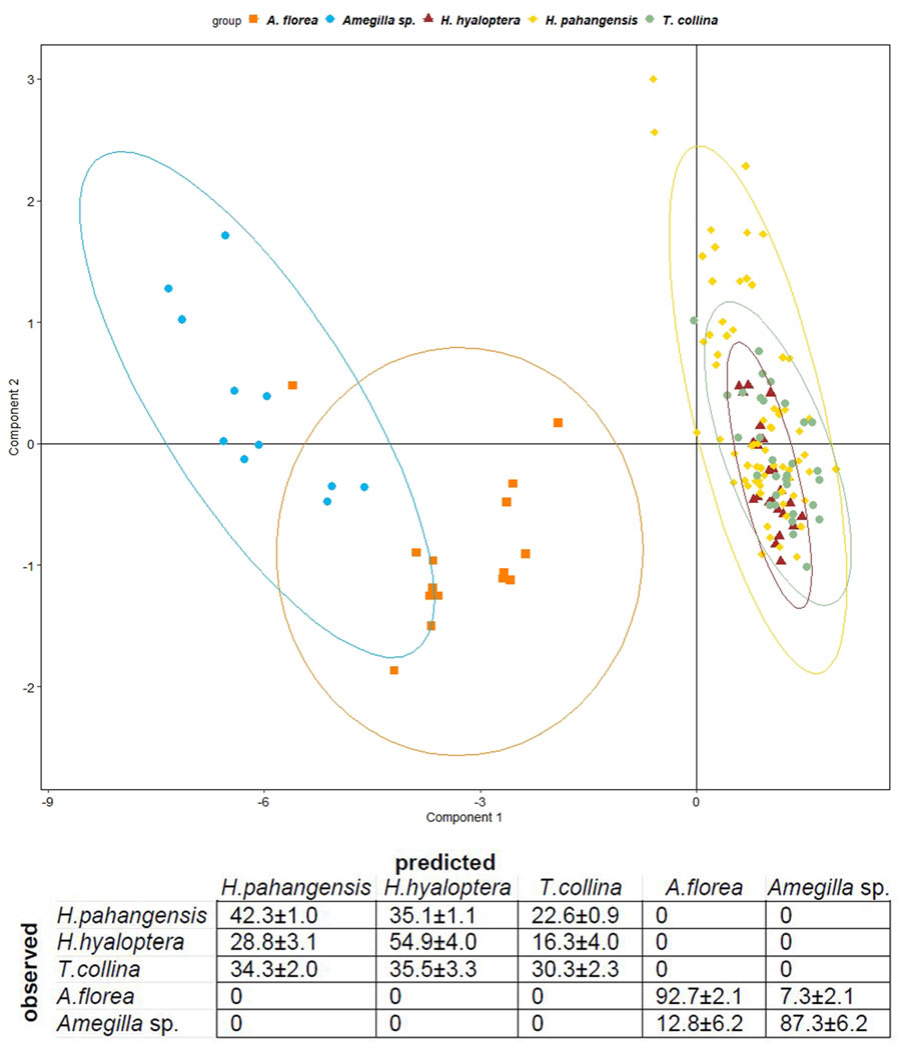
**A**. Representation of standardised components 1 and 2 of partial least squares discriminant analysis for models (*Tetragonilla collina*), mimics (*Heterosphecia pahangensis* and *H. hyaloptera*) and controls (*Apis florea* and *Amegilla* sp.). The first two components accounted respectively for 82% and 11 %. **B**. Contingency table showing percentages (± SE) of buzz assignment for each species after full cross validation tests

The PLS-DA (Table 7 in Additional file 2) shows that buzzing sounds of clearwing moths and stingless bees largely overlap forming one group separated from the buzzes of dwarf honey bees and blue banded bees (Fig. 3 A).

Full cross validation indicates that buzzes of the model *T. collina* are confused with the sounds of clearwing moths in about 70 % of all instances. *T. collina* and the clearwing moths are never incorrectly assigned to *A. florea* and *Amegilla* sp. bees (Fig. 3B). *A. florea* and *Amegilla* sp. emit such highly species-specific sounds that they can hardly be misidentified, i.e. 93 % and 87 % of sounds, respectively, are correctly assigned and only 7–13 % of cases are confused between the two species (Fig. 3B). A high proportion of variance is explained by the first component (nearly 82 %) and, cumulatively, over 93 % by the first two components. The observed variability for the first component is best explained by the fundamental frequency and its two harmonics (see Table 7 in Additional file 2 for details) followed by the third frequency quartile. Frequency components and standard deviation of the frequency spectrum affect the variance of the second component.

### Wingbeat frequencies

Wingbeat frequencies calculated from slow-motion videos vary among the five species (F_41,4_ = 176.8, *p* < 0.001), but do not differ between clearwing moths and their model bee, *T. collina* (Fig. 2; Table 6 in Additional file 2). Average wingbeat frequency (*H. pahangensis* 105.5–129 Hz, *H. hyaloptera* 105–123 Hz, *T. collina* 108–134.5 Hz, *A. florea* 166.5–250 Hz and *Amegilla* sp. 215.5–263.5 Hz) mirror and overlay the fundamental frequency range measured from recordings of their sounds.

## Discussion

Our findings reveal that *H. pahangensis*, besides morphologically and behaviorally imitating its model [15], also uses acoustic mimicry. We showed that the buzzing sounds produced in flight by both clearwing moth species, *H. pahangensis* and *H. hyaloptera*, highly resemble those emitted by *Tetragonilla* bees. The differences found between the clearwing moths’ buzz and those of the two other sympatric bees suggest that *H. pahangensis* and *H. hyaloptera* are specialised acoustic mimics. By vibrating their wings, models and mimics produce similar sounds despite anatomical differences, in particular in the size and shape of fore- and hindwing. In *T. collina*, the forewings are much bigger than the hindwings, while in clearwing moths it is the other way around: the forewings are very narrow, whereas the hindwings are wider (Supplementary Fig. 1 in Additional file 2). However, when both wings are considered together, the general similarity in shape and area is evident. Clearwing moths have the most sophisticated wing coupling mechanism in all Lepidoptera [22]: apart from the frenulum protruding from the hindwing and held by the reticulum on the forewing, the edges of both wings are rolled (see Figs. 3–4 in [16]). Also bees keep fore- and hindwings together in flight (see video [Additional file 1 TC 01:50–02:16]). Therefore, both in models and mimics the two pairs of wings act as one plane and together contribute to producing the buzzing sound.

In contrast, buzzing sounds emitted by the two control species of bees differ significantly from *T. collina* and the clearwing moths but also from each other. This is probably due to marked differences in their body size and flight behaviour. The slender *A. florea* worker has a body length of 7–10 mm with forewing length of 6.1–6.9 mm [23], whereas the more robust *Amegilla* sp.’ body length is 10.9–13.9 mm, with forewing length of 7.65–8.75 mm [24]. In addition, *Amegilla* sp. is a markedly faster and more manoeuvrable flyer than *A. florea* (Skowron Volponi & Volponi, pers. obs.).

Acoustic mimicry of flying hymenopterans has barely been studied, with only a few attempts performed on hoverflies (Diptera: Syrphidae) and none on clearwing moths. To the best of our knowledge, only one other species of Sesiidae has been recorded. Teasdale [25] registered the wing-fanning sounds of *Pennisettia marginata* Harris, 1839, testing their potential role in sexual communication. Sound was proven not to play part in male/female interactions and additionally the author briefly mentioned that sound frequencies of *P. marginata* do not correspond to the frequencies of their assumed mimicry models, which were not recorded in the study. However, the recorded sounds were not of sesiids in free flight. Moreover, only common *Vespula* and *Dolichovespula* species were referred to as potential models, although it has never been assessed if these are in fact the wasps mimicked by *P. marginata*. It is worth noting that fundamental sound frequencies of male *P. marginata* (112.0 ± 1.9 Hz) fell in the low end of the range of frequencies that we report here for both *Heterosphecia* species. Perhaps comparing the sounds of *P. marginata* with additional potential models would yield acoustical similarities. As far as studies of acoustic mimicry of hymenopterans in different groups of insects are concerned, Gaul [26] proved that the hoverfly *Spilomyia hamifera* Walker, 1849 has a remarkably similar wingbeat frequency to its visual model *Dolichovespula arenaria* Fabricius, 1775 (Hymenoptera: Vespidae), which corresponds to similar generated sounds. However, the insects were not recorded with microphones, and the author’s conclusions remain theoretical. Rashed *et al*. [27] compared the sounds made by tethered hoverflies, which were morphological mimics of hymenopterans, with sounds made by their models. Acoustic signals were recorded after imposing an attack simulated by squeezing the insects with forceps. No significant similarities between wasp and honeybee models and their mimics were found, but one of the tested hoverflies, a bumblebee mimic, produced similar sounds to a bumblebee considering the two tested acoustical parameters: fundamental frequency and the difference between the peak energy at the fundamental frequency and the peak energy at its harmonic. However, also honeybee- and wasp-like hoverflies emitted buzzes resembling those of bumblebees, suggesting that species considered in this study do not mimic specifically acoustic signals of their morphological models. Moore & Hassall [28] compared the sounds of tethered hoverflies and bumblebees before and after simulated attacks, but proved the existence of acoustic resemblance only in 2 out of 13 studied hoverfly species. However, tethering insects may alter results, while recording and comparing sounds of untethered, flying insects in their habitat would provide much more reliable or biologically meaningful conclusions. Immobilised insects may produce altered sounds as compared to those of unmanipulated ones. Rashed *et al*. [27] assumed that it is the alarm signals that are mimicked, but we must consider that bees and wasps do not only produce sounds upon predator attack but „buzz” during normal flight.

The existence of mimics producing vibroacoustic cues which resemble those emitted by a defended model has rarely been demonstrated, primarily in moths. Recently, however, Pekár and colleagues [29] showed that the spider *Palpimanus gibbulus* (Araneae: Palpimanidae) acts as a Batesian mimic by emitting stridulations similar to those produced by co-occurring mutillid wasps (Hymenoptera: Mutillidae). Predators such as other spiders or geckoes avoid or drop both stridulating mimics (*Palpimanus* spiders) and models (wasps). In this case, stridulations are not only alarming cues but can be considered as true aposematic signals emitted before contact with a predator.

The clearwing moth *H. pahangensis*, with its narrow, transparent wings, tufts of elongated scales on the hind legs resembling pollen baskets and bright bands on the abdomen, was previously known to have an overall bee-like appearance, even if it is not a perfect visual mimic of *T. collina*. It is more accurate in imitating the way bees fly (tracing trajectories highly similar to those of *T. collina*, [15]) and we now show that the resemblance of sounds produced in flight is equally accurate. Interestingly, *H. hyaloptera*, with a yellow band on the abdomen, also sounds like *T. collina*, but externally resembles the black stingless bee to an even lesser extent than *H. pahangensis*. In flight, however, when details of morphology are hard to discern, both studied clearwing moths are nearly indistinguishable from small, black bees (Skowron Volponi & Volponi, pers. obs.). Members of the family Sesiidae represent one of the most sophisticated mimics in the insect world, combining similarity to their hymenopteran models in size, shape, pattern, locomotion behaviour and, as we now show, sounds produced in flight. Such examples of multimodal signalling raise the question of how complex does a potential prey’s warning display have to be for a predator to abandon the idea of attack? Rothschild [30] argued that it is sufficient to trigger a predator’s bad memory of a previous unpleasant encounter and those several seconds of hesitation might allow the mimic to survive (Rothschild named this *aide memoire mimicry*). In terms of acoustic mimicry this could mean that the sound produced by a mimic does not have to be exactly the same as the model’s in order to deceive a predator, but should show similarities in one of its main features [31]. Since both predator and prey are likely to be in motion, acoustical features which are fixed regardless of distance, such as the fundamental frequency, are more likely to convey a true signal in a mimic-model system. Parameters connected to power and intensity, on the other hand, reach the predator in a varying degree depending on the distance from the sound source and often deliver accurate information on the spatial position rather than on the prey’s identity (see the moths and bats ultrasound signalling system first described by Roeder [32]). Drawing conclusions concerning the potential existence of acoustic mimicry based on intensity characteristics measured from an immobilised insect could be misleading. In our study, the fundamental frequency and its two harmonics best differentiated the mimic-model group from the control species and it is highly probable that these would also be the most meaningful features (conveying information about the identity of the emitting source) if placed in a biological context.

The complexity of signals involved in the mimicry of clearwing moths and bees could have several explanations. Morphological, behavioural and acoustic mimicry could serve in different situations, e.g. resembling flight trajectories discourages an avian predator observing a clearwing moth from a distance, whereas buzzing sounds and details of morphology provide benefit at close range. It is also possible that distinct signals have an effect on different recipients, e.g. acoustic signalling could be meant to disguise clearwing moths from potentially aggressive bees (and perhaps also wasps) in foraging groups, instead of from birds. *H. pahangensis* and *H. hyaloptera* puddle for salt at exactly the same locations, even the same pebble, as *T. collina* and other bees and, less frequently, wasps (Skowron Volponi & Volponi, pers. obs.; shown in additional video [see Additional file 1 TC 00:39–00:59]), whereas numerous other lepidopterans puddling for salt on the same riverbank usually keep their distance from aggregations of hymenopterans. Given that bees communicate both acoustically and chemically [33], it is possible that chemical mimicry is also part of the elaborate masquerade of *Heterosphecia* species – an interesting direction for future studies. Multimodal mimicry in the *Heterosphecia*-*Tetragonilla* system may have evolved due to selective pressure imposed by the presence of multiple predators, which can differ in several biological traits, e.g. hunting strategy, period of activity, prey detection abilities. Visual cues are only effective in high visibility conditions. At dusk, for instance, acoustic or olfactory signals might become more informative for predators tracking their prey [34]. Unpalatable tiger moths are aposematically coloured to warn birds about their toxicity and additionally produce ultrasonic clicks aimed at bats. Interestingly, they cease to emit ultrasounds in the spring when bats are less active [35]. In clearwing moths, predation pressure from lizards, spiders or other insectivorous invertebrates may have led to the development of acoustic and/or chemical signals, whereas morphological and locomotor mimicry may be more effective against birds. To reveal whom acoustic signalling is aimed at, it would be highly informative to perform playback experiments with potential predators with different hunting strategies. The use of engineered acoustic signals in these bioassays (e.g. with some frequency components suppressed) would additionally shed light on which parameter is most meaningful in vehiculating the message to a predator. Dissecting this information would be crucial to driving further research aimed at assessing the existence of acoustic mimicry by focussing not necessarily on the overall signal, but on its most informative components.

Clearwing moths displaying hymenopteran signals are, following a spot-on formulation by Vane-Wright [36], parasites of the communication of bees and wasps with predators. The whole picture of their complex mimicry is yet to be revealed.

## Conclusions

The clearwing moths *H. pahangensis* and *H. hyaloptera* produce similar sounds in flight to the stingless bee *T. collina*, which should be an advantageous feature in predator avoidance. These acoustical similarities, their morphological resemblance to *T. collina*, as well as the imitation of flight trajectories of this stingless bee [15], indicate that clearwing moths display multimodal mimicry of their evolutionary models, with additional levels of the phenomenon awaiting discovery. Unravelling the array of modalities in signalling will be crucial to achieving a clearer view of the adaptive role of multimodal communication.

Remarkable interordinal sound resemblances between diurnal moths and bees could act as reinforcing signals in multimodal communication of insects [31] in their endless struggle to survive dangerous predator-prey interactions.

## Methods

### Sound and video recordings

Two species of clearwing moths (Lepidoptera: Sesiidae), and three species of sympatric bees (each belonging to a different Apidae tribe) were recorded in Malaysian (Perak and Pahang states) and Thai (Phetchaburi and Sa Kaeo provinces) rainforests while puddling on moist substrate of unpaved forest roads and river banks. Both studied Sesiidae species are rare, occur only seasonally in specific habitats, favourable weather conditions (hot sunny days with no rain) and remain in a given puddling location for no longer than several hours. *H. hyaloptera* is almost always observed individually (a maximum of three individuals was seen simultaneously and a total of 15 individuals over two expeditions to Thailand), whereas *H. pahangensis* can occasionally be observed in higher numbers (tens of individuals) but such peak occurrences only last one-two days (Skowron Volponi & Volponi, pers. obs. over seven years of studying this species). The model bee *T. collina* was very abundant and often encountered in groups, *A. florea* was observed in fewer locations but also in high numbers, whereas *Amegilla* sp. always occurred individually (understandably, as it is a solitary bee). Recordings were performed with a Sennheiser ME2 lavalier microphone (sensitivity: 20 mV/Pa; frequency response: 30-20000 Hz). The microphone, placed on a long stick, was connected to a Sennheiser Evolution G2 wireless system attached to a TASCAM DR-60D MKII linear PCM recorder with headphones allowing for real-time control of the recordings. A leaf was attached in proximity to the microphone for camouflage (Additional file 1 TC 01:00–01:07) and this highly improved our success in approaching insects without startling them. All insects were recorded in free, undisturbed flight without catching or handling them to ensure that the registered sounds were not alarm signals. Tethering small and extremely delicate clearwing moths would not be feasible, as their scale-covered bodies are easily damaged upon handling the insects. Recordings were initiated only when we heard the buzzing sound through the headphones, which indicated close proximity to the insect.

After verifying the quality of the obtained recordings, the following number of audio files was selected for further analysis: *Heterosphecia pahangensis* (n = 15; behavioural and morphological mimic, putative acoustic mimic), *H. hyaloptera* (n = 5; morphological mimic, putative acoustic mimic), *Tetragonilla collina* (n = 6; model) and two control species, *Apis florea* (n = 3) and *Amegilla* sp. (n = 2). Each audio file represents a separate individual. The *Amegilla* group of Southeast Asia is a complex of highly similar species distinguished by minor morphological features. The recorded species is most probably *Amegilla zonata* Linnaeus, 1758 but since specimens were not collected, definite identification could not be carried out and it is thus reported here as *Amegilla* sp.

Audio recordings were analysed and filtered with PRAAT 6.1.0.9. The pass Hann band with a smoothing level of 100 Hz was used to select frequency ranges in which the studied signals are audible and exclude vocalisations produced by other animals, e.g. birds, emitted at higher frequencies than the buzzing sounds of the studied species. Out of every recording, five units with a duration of 0.3 s were selected for analysis. Eight acoustic parameters were calculated for the first three frequency components: fundamental frequency or pitch (Hz), frequency of the first and second component above the pitch (Hz), dominant frequency (Hz), first, second and third frequency quartiles (Hz), standard deviation of the frequency spectrum (Hz), (see [37] and [38] for detailed descriptions of the sound parameters). Because insects were not tethered during recordings but were flying freely, we decided not to include any parameter dependent on the distance of the sound source from the microphone, namely sound intensity, amplitude, power and energy, in the statistical analyses.

Slow-motion (500, 800, 960 and 1000 frames per second) videos of insects in free flight were made with Sony RX10 II, Sony RX10 III or Sony NEX-FS700 cameras. Wingbeat frequencies were determined from slow-motion videos by counting the number of frames required to complete a wingbeat. At least 11 wingbeats were analysed per video and the mean wingbeat frequency was calculated.

Audio and video recordings were made with temperatures ranging from 28 to 32 °C in Malaysia and from 29 to 34 °C in Thailand and humidity from 68 to 84 % in Malaysia and 48–53 % in Thailand. These abiotic parameters were measured with an electronic thermo-hygrometer placed in the shade.

### Statistical analysis

To test for normality, density and quantile-quantile plots were examined and Shapiro-Wilk tests applied. According to data distribution, each retained acoustic parameter was tested for differences among the studied species through likelihood ratio tests using a linear mixed model with species as a fixed factor and individual as a random factor followed by Tukey’s tests, or Kruskal Wallis rank sum tests followed by Tukey and Kramer (Nemenyi) tests [39]. A one-way ANOVA test followed by a Tukey test was applied to test for differences between species in means of wingbeat frequencies calculated from slow-motion videos. Correlation between sound variables was assessed by calculating the Pearson correlation coefficient. Variables with values greater than 0.75 were considered strongly correlated and excluded from the multivariate analysis. To extract groups of sounds produced by mimics and models, as well as specify components that best explain variability between groups and the contribution of each audio parameter in their differentiation, we performed a partial least square discriminant analysis (PLS-DA). PLS-DA, implemented in the “mixOmics” R package [40], is a supervised pattern recognition technique which is largely unaffected by co-variance among acoustic parameters and small ratio between cases (samples) and variables (sounds) [41]. Moreover, a full cross validation (leave-one-out) test based on Mahalanobis distance was performed using the predict function to get a contingency table (confusion matrix) of posterior-predicted/observed membership of all samples. The average (± SE) proportions of assignment for each species were based on 999 simulations. Discriminant sound parameters responsible for species grouping were identified according to their influence on the projection (VIP) parameter. After calculating VIP scores for all spectrum variables, we then retained those with VIP values > 1 [42].

Statistical analyses were performed and plots drawn in R Studio version 1.2.5033 (R Development Core Team, 2020).

## Supplementary Information

**Additional file 2: Tables S1-S8** showing results of multiple comparisons of means of the tested acoustical parameters and Variable importance in projection (VIP) scores for the first two components obtained from the PLS-DA analysis. Supplementary **Figure S1** showing fore- and hindwing contours of the two clearwing moth species (mimics), *H. pahangensis* and *H. hyaloptera*, of the bee *T. collina* (model) and of *A. florea* bee, used as control in our work.

**Additional file 3.** Representative audio recording of *Heterosphecia pahangensis* in flight.

**Additional file 4.** Representative audio recording of *Heterosphecia hyaloptera* in flight.

**Additional file 5.** Representative audio recording of *Tetragonilla collina* in flight.

**Additional file 6.** Representative recording of *Apis florea* in flight.

**Additional file 7.** Representative recording of *Amegilla* sp. in flight.

**Additional file 8. **Results of acoustic calculations of every analysed individual in Hertz. Abbreviations: 3rdQ – third frequency quartile, StDev - standard deviation of the frequency spectrum, Fpeak – dominant frequency, F0peak – fundamental frequency, F1peak – frequency of the first component above the pitch, F2peak - frequency of the second component above the pitch.

**Additional file 9. **Mean wingbeat frequencies (Hz) calculated from slow-motion videos of every studied species.

## Data Availability

The datasets supporting the conclusions of this article are included within the article and its additional files.
