# Supplementary material for: Southeast Asian clearwing moths buzz like their model bees
Source: Front Zool. 2021 Jul 7;18:35. doi: 10.1186/s12983-021-00419-8 (PMC8262067; doi:10.1186/s12983-021-00419-8)
Supplement: Supplementary file 2 — Additional file 2: Tables S1-S8 showing results of multiple comparisons of means of the tested acoustical parameters and Variable importance in projection (VIP) scores for the first two components obtained from the PLS-DA analysis. Supplementary Figure S1 showing fore- and hindwing contours of the two clearwing moth species (mimics), H. pahangensis and H. hyaloptera, of the bee T. collina (model) and of A. florea bee, used as control in our work. [file 12983_2021_419_MOESM2_ESM.docx]

**Southeast Asian clearwing moths buzz like their model bees**

**Additional file 2**

**Table 1.** Multiple comparisons of means of dominant frequency by species using Tukey’s test presented significant differences (*p* < 0.05, indicated by an asterisk) between pairs of species. Tukey’s test was performed under the significant result of the likelihood-ratio test comparing a null model with a model with species as fixed factor and individual as random factor, χ^2^*=*115.13, *p* < 0.001.

| Pairwise comparisons | Mean difference | Standard error | z value | *p* value |
| --- | --- | --- | --- | --- |
| *H. pahangensis - H. hyaloptera* | -0.35 | 7.7877 | -0.045 | 1.000 |
| *H. pahangensis - T. collina* | -5.09 | 7.5292 | -0.677 | 0.959 |
| *H. pahangensis - A. florea* | -96.27 | 9.3116 | -10.338 | < 0.001* |
| *H. pahangensis - Amegilla* sp. | -334.48 | 10.7601 | -31.086 | < 0.001* |
| *H. hyaloptera - T. collina* | -5.45 | 9.2511 | -0.589 | 0.975 |
| *H. hyaloptera - A. florea* | -95.92 | 10.7518 | -8.921 | < 0.001* |
| *H. hyaloptera - Amegilla* sp*.* | -334.13 | 12.0280 | -27.779 | < 0.001* |
| *T. collina - A. florea* | -101.36 | 10.5660 | -9.593 | < 0.001* |
| *T. collina - Amegilla* | -339.58 | 11.8622 | -28.627 | < 0.001* |
| *A. florea - Amegilla* | 238.21 | 13.0664 | 18.231 | < 0.001* |

**Table 2.** Multiple comparisons of means of fundamental frequency by species using Tukey’s test presented significant differences (*p* < 0.05, indicated by an asterisk) between pairs of species. Tukey’s test was performed under the significant result of the likelihood-ratio test comparing a null model with a model with species as fixed factor and individual as random factor, χ^2^*=*124.31, *p* < 0.001.

| Pairwise comparisons | Mean difference | Standard error | z value | *p* value |
| --- | --- | --- | --- | --- |
| *H. pahangensis - H. hyaloptera* | -0.40 | 3.1467 | -0.126 | 1.000 |
| *H. pahangensis - T. collina* | -5.15 | 2.9994 | -1.716 | 0.411 |
| *H. pahangensis - A. florea* | -96.19 | 3.8142 | -25.218 | < 0.001* |
| *H. pahangensis - Amegilla* sp. | -133.75 | 4.4825 | -29.839 | < 0.001* |
| *H. hyaloptera - T. collina* | -5.54 | 3.7137 | -1.492 | 0.554 |
| *H. hyaloptera - A. florea* | -95.79 | 4.3981 | -21.780 | < 0.001* |
| *H. hyaloptera - Amegilla* sp. | -133.35 | 4.9888 | -26.731 | < 0.001* |
| *T. collina - A. florea* | -101.33 | 4.2939 | -23.599 | < 0.001* |
| *T. collina - Amegilla* | -138.90 | 4.8972 | -28.363 | < 0.001* |
| *A. florea - Amegilla* | 37.56 | 5.4345 | 6.912 | < 0.001* |

**Table 3.** Multiple comparisons of means of first frequency component by species using Tukey’s test presented significant differences (*p* < 0.05, indicated by an asterisk) between pairs of species. Tukey’s test was performed under the significant result of the likelihood-ratio test comparing a null model with a model with species as fixed factor and individual as random factor, χ^2^*=*119.66, *p* < 0.001.

| Pairwise comparisons | Mean difference | Standard error | z value | *p* value |
| --- | --- | --- | --- | --- |
| *H. pahangensis - H. hyaloptera* | -0.85 | 6.7207 | -0.127 | 1.000 |
| *H. pahangensis - T. collina* | -11.44 | 6.3951 | -1.789 | 0.367 |
| *H. pahangensis - A. florea* | -194.68 | 8.1558 | -23.871 | < 0.001* |
| *H. pahangensis - Amegilla* sp. | -261.21 | 9.5988 | -27.212 | < 0.001* |
| *H. hyaloptera - T. collina* | -12.30 | 7.9266 | -1.551 | 0.515 |
| *H. hyaloptera - A. florea* | -193.83 | 9.4049 | -20.610 | < 0.001* |
| *H. hyaloptera - Amegilla* sp. | -260.35 | 10.6804 | -24.377 | < 0.001* |
| *T. collina - A. florea* | -206.13 | 9.1751 | -22.466 | < 0.001* |
| *T. collina - Amegilla* | -272.65 | 10.4786 | -26.020 | < 0.001* |
| *A. florea - Amegilla* | 66.52 | 11.6371 | 5.717 | < 0.001* |

**Table 4.** Multiple comparisons of means of second frequency component by species using Tukey’s test presented significant differences (*p* < 0.05, indicated by an asterisk) between pairs of species. Tukey’s test was performed under the significant result of the likelihood-ratio test comparing a null model with a model with species as fixed factor and individual as random factor, χ^2^*=*120.07, *p* < 0.001.

| Pairwise comparisons | Mean difference | Std. error | z value | *p* value |
| --- | --- | --- | --- | --- |
| *H. pahangensis - H. hyaloptera* | -8.62 | 10.059 | -0.857 | 0.908 |
| *H. pahangensis - T. collina* | -13.18 | 9.554 | -1.380 | 0.628 |
| *H. pahangensis - A. florea* | -288.42 | 12.223 | -23.597 | < 0.001* |
| *H. pahangensis - Amegilla* sp. | -395.05 | 14.408 | -27.420 | < 0.001* |
| *H. hyaloptera - T. collina* | -21.80 | 11.856 | -1.839 | 0.338 |
| *H. hyaloptera - A. florea* | -279.80 | 14.095 | -19.850 | < 0.001* |
| *H. hyaloptera - Amegilla* sp. | -386.43 | 16.027 | -24.111 | < 0.001* |
| *T. collina - A. florea* | -301.60 | 13.739 | -21.952 | < 0.001* |
| *T. collina - Amegilla* | -408.23 | 15.715 | -25.978 | < 0.001* |
| *A. florea - Amegilla* | 106.63 | 17.466 | 6.105 | < 0.001* |

**Table 5.** Multiple comparison of means of third frequency quartile by species. The Kruskal-Wallis rank sum test indicated significance: χ^2^(4) = 60.509*, p* < 0.001. The Tukey and Kramer (Nemenyi) test showed significant differences (*p* < 0.05, indicated by an asterisk) between pairs of species.

|  | *H. pahangensis* | *H. hyaloptera* | *T. collina* | *A. florea* |
| --- | --- | --- | --- | --- |
| *H. hyaloptera* | χ^2^= 1.681; *p* = 0.760 |  |  |  |
| *T. collina* | χ^2^= 0.776; *p* = 0.980 | χ^2^= 0.781; *p* = 0.980 |  |  |
| *A. florea* | χ^2^= 7.195; *p* < 0.001* | χ^2^= 7.447; *p*  < 0.001* | χ^2^= 6.885; *p*  < 0.001* |  |
| *Amegilla* sp. | χ^2^= 7.549; *p* < 0.001* | χ^2^= 7.839; *p* < 0.001* | χ^2^= 7.331; *p* < 0.001* | χ^2^= 1.0716; *p* = 0.940 |

**Table 6.** Multiple comparison of means of standard deviation of spectrum by species. The Kruskal-Wallis rank sum test indicated significance: χ^2^(4) = 37.525*, p* < 0.001. The Tukey and Kramer (Nemenyi) test showed significant differences (*p* < 0.05, indicated by an asterisk) between pairs of species.

|  | *H. pahangensis* | *H. hyaloptera* | *T. collina* | *A. florea* |
| --- | --- | --- | --- | --- |
| *H. hyaloptera* | χ^2^= 3.180; *p* = 0.162 |  |  |  |
| *T. collina* | χ^2^= 2.742; *p* = 0.300 | χ^2^= 0.440; *p* = 1.008 |  |  |
| *A. florea* | χ^2^= 2.997; *p* = 0.212 | χ^2^= 4.901; *p* < 0.001* | χ^2^= 4.597; *p* = 0.010* |  |
| *Amegilla* sp. | χ^2^= 5.863; *p* < 0.001* | χ^2^= 7.308; *p* < 0.001* | χ^2^= 7.060; *p* = *p* < 0.001* | χ^2^= 2.684; *p* = 0.318 |

**Table 7.** Multiple comparisons of means of wingbeat frequency by species using Tukey’s HSD test presented significant differences (*p* < 0.05, indicated by an asterisk) between pairs of species. Tukey’s test was performed under the significant result of the one-way ANOVA test, F*=*176.8, *p* < 0.001.

| Pair of species | Mean difference | *p* value |
| --- | --- | --- |
| *H. pahangensis - H. hyaloptera* | 1.53 | 0.996 |
| *H. pahangensis - T. collina* | 4.02 | 0.962 |
| *H. pahangensis - A. florea* | -67.39 | < 0.001* |
| *H. pahangensis - Amegilla* sp. | -119.43 | < 0.001* |
| *H. hyaloptera - T. collina* | 5.54 | 0.895 |
| *H. hyaloptera - A. florea* | -68.92 | < 0.001* |
| *H. hyaloptera - Amegilla* sp. | -120.96 | < 0.001* |
| *T. collina - A. florea* | -63.38 | < 0.001* |
| *T. collina - Amegilla* sp. | -115.42 | < 0.001* |
| *A. florea - Amegilla* sp. | 52.04 | < 0.001* |

**Table 8.** Variable importance in projection (VIP) scores for the first two components obtained from the PLS-DA analysis.

| Variable | 1^st^ component | 2^nd^ component |
| --- | --- | --- |
| Fundamental frequency | 1.116 | 1.031 |
| First frequency component | 1.112 | 1.040 |
| Second frequency component | 1.117 | 1.040 |
| Dominant frequency | 0.918 | 0.868 |
| Third frequency quartile | 1.064 | 0.973 |
| SD of frequency spectrum | 0.545 | 1.036 |


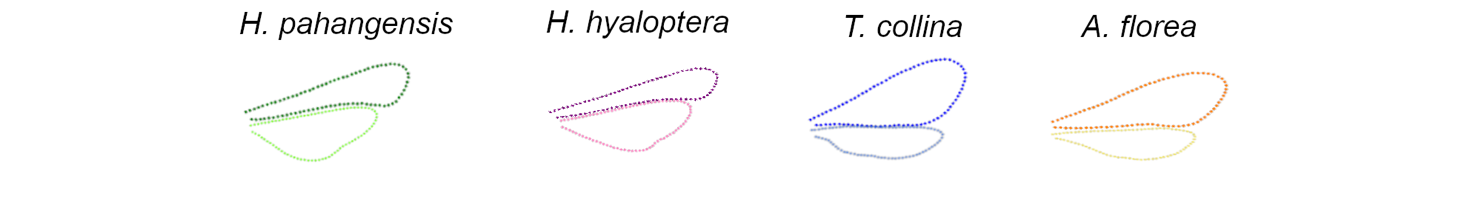
**Supplementary Figure 1.** Wing contour of the fore- and hindwings of the two clearwing moth species (mimics), *H. pahangensis* and *H. hyaloptera*, of the bee *T. collina* (model) and of *A. florea* bee, used as control in our work.
